# Disorganized Attachment pattern affects the perception of Affective Touch

**DOI:** 10.1038/s41598-020-66606-5

**Published:** 2020-06-15

**Authors:** Grazia Fernanda Spitoni, Pietro Zingaretti, Guido Giovanardi, Gabriella Antonucci, Gaspare Galati, Vittorio Lingiardi, Gianluca Cruciani, Giulia Titone, Maddalena Boccia

**Affiliations:** 1 Department of Dynamic and Clinic Psychology, Sapienza University of Rome, Rome, Italy; 2 Cognitive and Motor Rehabilitation and Neuroimaging Unit, IRCCS Fondazione Santa Lucia), Rome, Italy; 3 Villa von Siebenthal Hospital, Genzano di Roma, Italy; 4 Department of Psychology, University of Campania, Luigi Vanvitelli, Caserta Italy; 5 Department of Psychology, Sapienza University of Rome, Rome, Italy; 6 Department of Psychology, PhD Program in Behavioural Neuroscience, Sapienza University of Rome, Rome, Italy; 7 Utrecht University, Faculty of Social and Behavioural Sciences, Experimental Psychology, Utrecht, The Netherlands

**Keywords:** Human behaviour, Social neuroscience, Social neuroscience, Human behaviour

## Abstract

Touch, such as affective caress, can be interpreted as being pleasant. The emotional valence that is assigned to touch is related to certain bottom-up factors, such as the optimal activation of C-tactile (CT) afferents. Tactile processing with a hedonic or emotional component has been defined as affective touch—a component that CT fibers are likely to convey. Tactile deficiencies are frequent in the psychiatric population but also in healthy people with disorganized attachment; accordingly, it is likely that affective difficulties in adults with disorganized attachment are reflected in altered perception of affective touch. To test this hypothesis, we combined methods from clinical psychology, psychophysics, and neuroimaging. We found that people with a history of traumatic parental bonds and a disorganized attachment pattern perceive a “caress-like” stimulus as being unpleasant, whereas participants with organized attachment consider the same tactile stimulation to be pleasant. Further, unlike in organized adults, the responses of disorganized adults to CT and non-CT stimulation activated limbic and paralimbic structures in a fight-or-flight manner, suggesting that early experiences with parental deficiencies shape the physiological responses of peripheral CT fibers and central nervous networks.

## Introduction

In humans, touch is the first sensory system to develop^1,2^. The earliest sensations that we experience are tactile. At 12 weeks of gestation, the cutaneous receptors and somatosensory functions have matured^3^, and the fetus is able to make movements when their lips are touched^4^; in contrast, other sensory modalities, such as hearing and vision, develop later. The early emergence of tactile functions in fetal growth suggests that its initial tactile experiences are crucial for the development and maturation of an organism. This hypothesis has been demonstrated in several developmental pathways, from biological growth^1^ and psychological maturity^5^ to achievements in social skills^6^. Sigmund Freud, whose background was in neurophysiology, defined the skin as the “first psychological organ”^7^.

Despite the evidence that has posited the primacy of touch in human development, we owe the first attempts to link the sense of touch to social, cognitive, and affective domains to studies on animals, among which research by Harlow^8,9^ on rhesus monkeys remains a cornerstone. In his famous studies, Harlow demonstrated that infant rhesus monkeys preferred to cling to a surrogate wire mother that was covered in warm cloth than to one that provided milk but comprised only wires. Harlow also observed that on experiencing a sudden frightening stimulus, the cloth model was again preferred to the wire mother, wherein the monkeys sought immediate physical contact with the cloth model, after which their fear subsided. Based on these findings, Harlow suggested that the absence of comforting touch led to psychological stress in the monkeys. Likely, these studies provided the seminal evidence of the influential function of bodily contact in the development of attachment in infant monkeys.

In the attachment theory, Bowlby suggested that children enter the world biologically pre-programmed to form attachments with others, because this phenomenon will help them survive. According to Bowlby’s theory^10,11^, attachment behaviors are instinctive and are activated by any conditions that seem to threaten the achievement of proximity, such as separation, insecurity, and fear. Bowlby also documented that human children who are deprived of motherly contact often develop psychological problems.

In the past 3 decades, many authors have based their work on the extraordinary intuitions of Harlow and Bowlby. Since the 1990s, several authors used the sense of touch to further explore the theory of attachment. For example, Reite^12^ suggested that in humans, touch is fundamental, because it potentiates the formation of an affective relationship with the primary caregiver—primarily the mother—which in turn forms a “secure” base that facilitates the development of learning, emotional regulation, and social interactions. On similar premises, Anisfeld *et al*.^13^ found strong evidence that at age 13 months, increased physical contact promotes more secure attachments in infants. In another study on touch and attachment, Weiss *et al*.^14^ explored aspects of maternal touch and its ship with the security of attachment of a low-birth-weight infant at age 1 year. The results of this observational study showed that at 1 year, nurturing touch was associated with more secure attachment; the group also found that children whose mothers felt more secure about their own childhood experiences with touch were more likely to develop secure attachments. As expounded on in our discussion, these premises form a basis for understanding the relevance of the attachment patterns of infants and adults with regard to adult therapies^15^.

More recently, touch has been used to study the influence of attachment styles in the perception of physical pain^16^. Specifically, the authors examined whether various properties of touch modulate subjective and neural responses to pain with respect to individual attachment style. Notably, pleasant touch reduced the perception of pain in individuals with greater attachment anxiety and, conversely, increased pain in those with higher attachment avoidance. Further, certain self-harm behaviors, such as those observed in borderline personality disorders, implicate a link between attachment style and physical pain^17^.

In the aforementioned study, the experimenters referred to a specific tactile system, known as the C tactile (CT) system.

According to Olausson *et al*.^18^ the slow stimulation of hairy skin correlates frequently with the perception of pleasant sensations that strongly evoke a caress-like experience. McGlone *et al*.^19,20^ described a specialized neurophysiological system that mediates affective touch, rather than other properties of touch—as discussed, this system is called the CT afferent system. CT fibers are a population of unmyelinated, low-threshold, mechanoreceptive afferents that are exclusive to hairy skin and respond optimally to innocuous stimuli. They are activated most vigorously by subjectively rated pleasant stroking velocities and respond optimally to intermediate velocities of 1–10 cm/s. Slower or faster velocities result in suboptimal CT activation and yield lower pleasantness ratings^20^. Notably, the afferent C fiber family includes pleasant touch, as well as pain, temperature, and itch. Yet, the functional significance of the survival of a nociceptive system is unequivocal—it is more difficult to describe the evolutionary functions of the “affective receptors” that populate the hairy skin.

Why should a system that conveys hedonic feelings of touch have utility in the development of an organism? Clearly, there is not a sole answer to this question. When reviewing the principal features of the CT system, McGlone^20^ identified a fundamental function of pleasant touch—namely, reward. Indeed, CT fibers are triggered by caress-like stimuli, constituting a peripheral mechanism for signaling pleasant skin-to-skin contact in humans, which in turn promotes interpersonal touch and affiliative behavior. This psychobiological perspective appears to support the studies of Bowlby^10,11^, who suggested that the attachment system is activated when, after separation from the caregiver, the child seeks proximity to the parent or caregiver in the form of physical contact, which leads him to feel secure and safe.

The intrinsic bond between affective touch and psychological dimension implies that the former is altered in the case of psychological vulnerabilities—circumstances that have been examined in several studies. For example, Crucianelli *et al*.^21^ demonstrated that patients who are affected by anorexia nervosa perceive affective touch to be less pleasant than healthy controls. In a descriptive study, Croy *et al*.^22^ tested the change in affective touch in a large sample of psychotherapy outpatients who had a broad range of mental disorders (mood and affective, personality disorders, post-traumatic stress, and anxiety disorders). The authors found that patients rated touch as being generally less pleasant than controls, but notably, this effect was stronger in patients with personality disorders. Similarly, a recent study by Strauss and co-workers^23^ showed that female patients with PTSD as a result of violence and sexual abuse, rated several touch conditions (namely interpersonal and impersonal tactile stimulations) as less pleasant than controls did. In addition, unlike controls, patients rated affective touch as negative. All together these finding suggest that the interpersonal and social functions that are decoded by the affective touch system are traceable in adults who experience difficulties in relating to others in the affective domain and in impulse control.

These 2 difficulties characterize a specific attachment pattern: disorganized-unresolved. According to the literature on attachment, the disorganized-unresolved pattern triggers dissociated traumatic memories that are related to fearful or neglectful experiences of attachment^24^ and includes contradictory and dramatic expectations in relation to caregivers^25–27^. This pattern has been linked to emotional dysregulation, extreme behavioral reactions in stressful situations^24^, and frequently to self-harming conduct; it is overrepresented in clinical samples that are characterized by the phenomena of dissociation (eg, post-traumatic stress disorder) and dysregulation in impulse and emotion (eg, borderline personality disorder)^28^. Given these circumstances, it is likely that affective difficulties and impulse dyscontrol in adults with disorganized attachment (eg, self-cutting behaviors in patients with borderline personality disorders) are reflected in altered perception of affective touch.

Accordingly, the proposals in this work can be divided into 2 separate studies. In the first study, we examined whether people with disorganized attachment perceive affective touch as being less pleasant than those with organized attachment. Using the Adult Attachment Interviews as the standard assessment of attachment style, we predict that given the social and emotional function of affective touch and the affective difficulties that are experienced by persons with disorganized attachment, the perception of the pleasantness of touch is reduced in those with disorganized attachment. Consistently, we have found that persons with disorganized attachment rate affective stimulations as less pleasant than those with an organized attachment style.

In the second study, we determined the neural substrate of the altered perception of affective touch in people with disorganized attachment. Previous imaging studies of healthy people identified several areas of the brain that are involved in affective touch. Among them, the insular cortex is considered the primary neural target of the affective touch system^29–31^. Other areas include the supramarginal gyrus^32^, primary somatosensory cortex^33^, amygdala^34^, and orbitofrontal cortex^35^.

Here, we performed an fMRI study in which Affective and Non-affective stimulation was administered in pseudorandomized blocks. A subgroup of participants in Study 1 with organized and disorganized attachment o participated in the fMRI study. In a preliminary step, we expect to detect a general network of brain areas that are typically involved during Affective stimulation. In this network, we hypothesized that people with disorganized attachment, who preferred Non-affective stimulation (see results of Study 1), will show higher activation of the areas of the brain that process general emotional arousal, such as the limbic and paralimbic cortex, under

## Results

### Study 1

#### Attachment patterns do not affect discriminative touch performance and the self-report psychological scales

The dataset had no missing data. AAIs were coded as follows: 17 were classified as Disorganized/Unresolved (Disorganized Attachment = DA; 10 by females) and 46 were classified as Organized/Resolved (Organized Attachment = OA; 21 by females) with respect to attachment. Indices of disorganized/unresolved responses were related to experiences of loss, abuse, or both regarding primary caregivers (parents or grandparents).

Given the absence of sex distribution differences between DA and OA groups (*χ*^2^ = 1.37; *p* = 0.18), sex was not included as a covariate in the statistical analyses.

As expected, when OA participants were compared with DA participants no significant difference emerged in basic discriminative touch (acuity, sensitivity and thermal threshold) and in the self-report (PID-5 and SCL-90-R) with the exception in the Somatization scale where DA got higher scores then OA respectively (mean = 0,62 ± 0,42 VS mean 0,93 ± 0,42; (*t*_(61)_= 2.7; *p* < 0.008. *Cohen’s d* = 1.39).

The demographic, clinical information and two sample t Test are reported in Table 1. See also Table S1 in supplementary section for additional data.

**Table 1:** Pre-existing group differences (means and standard deviations) in the demographic and clinical scales of the study.

	Organized (N = 46; 21F, 25M)		Disorganized (N = 17; 10F, 7M)			
	Min-Max	Mean (Std.Dev.)	Min-Max	Mean (Std.Dev.)	Student t	Cohen’s *d*
*Age (Years)*	19–50	29,02 (8,01)	19–56	31,59 (11,08)	−1,01, *NS*	−0,266
*Education(years)*	8–18	16,3 (7,71)	8–18	15,9 (7,5)	−1,16, NS	−0,34
*PID – Negative Affect*	0,12 – 2,19	1,08 (0,53)	0,12–2,19	1,2 (0,61)	−0,7 NS	-0,208
*PID – Detachment*	0,07–2,30	0,69 (0,41	0,00–2,21	0,7 (0,62)	−0,08, *NS*	−0,019
*PID – Antagonism*	0,04–1,76	0,61 (0,47)	0,07–1,96	0,7 (0,48)	−0,68, *NS*	−0,189
*PID – Disinhibition*	0–2,01	0,78 (0,47)	0,00–1,83	0,9 (0,54)	−0,86, *NS*	−0,237
*PID – Psychoticism*	0–1,71	0,7 (0,56)	0,07–2,32	0,9 (0,65)	−1,21, *NS*	−0,330
*SCL-90-R (global score)*	0,05–2,3	0,66 (0,52)	0,06-2,28	0,9 (0,58)	−1,53, *NS*	−0,432
*SCL-Somatization*	0–3,08	0,62 (0,65)	0–2,17	0,93 (0,56)	−1,68, *NS*	−0,51
*SCL-Obsessive-Comp*.	0–3,00	0,85 (0,65)	0–2,40	1,18 (0,76)	−1,64, *NS*	−0,466
*SCL-Interpersonal sens*.	0–2,56	0,61 (0,60)	0–3,89	0,95 (0,91)	−1,59, *NS*	−0,441
*SCL-Depression*	0–2,85	0,80 (0,66)	0–2,69	1,09 (0,82)	−1,37, *NS*	*−0,389*
*SCL- Anxiety*	0–3,00	0,70 (0,68)	0–2,30	0,89 (0,59)	−0,94, *NS*	−0,305
*SCL- Hostility*	0–3,33	0,73 (0,74)	0–2,50	0,83 (0,71)	−0,50, *NS*	−0,137
*SCL-Phobic anxiety*	0–1,29	0,19 (0,32)	0–2,71	0,30 (0,63)	−0,86, *NS*	−0,22
*SCL- Paranoid ideation*	0–2,83	0,80 (0,69)	0–2,67	1,16 (0,70)	−1,753, *NS*	−0,517
*SCL-Psychoticism*	0–2,40	0,39 (0,46)	0–1,70	0,55 (0,59)	−1,05, *NS*	−0,302
*VonFrey (gr)*	1,65 – 3,84	3,01 (0,63)	1,65 – 3,84	3,09 (0,62)	10,44, *NS*	−0,128
*2PD (cm)*	1,5–9	4,09 (1,68)	2–6	3,73 (0,97)	0,83, *NS*	0,262
*TS (°C)*	41,5 – 49,2	44,6 (1,64)	41,5 – 44,8	44,8 (1,46)	−0,39, *NS*	−0,116

Note: OA = Organized Attachment; DA = Disorganized Attachment; PID = Personality Inventory for DSM-5; SCL-90-R = Symptom Checklist-90-R; 2PD = Two Point Discrimination; TS = Thermal sensitivity.

#### Comparing organized/disorganized in perceiving the pleasantness of touch

**Figure 1:**
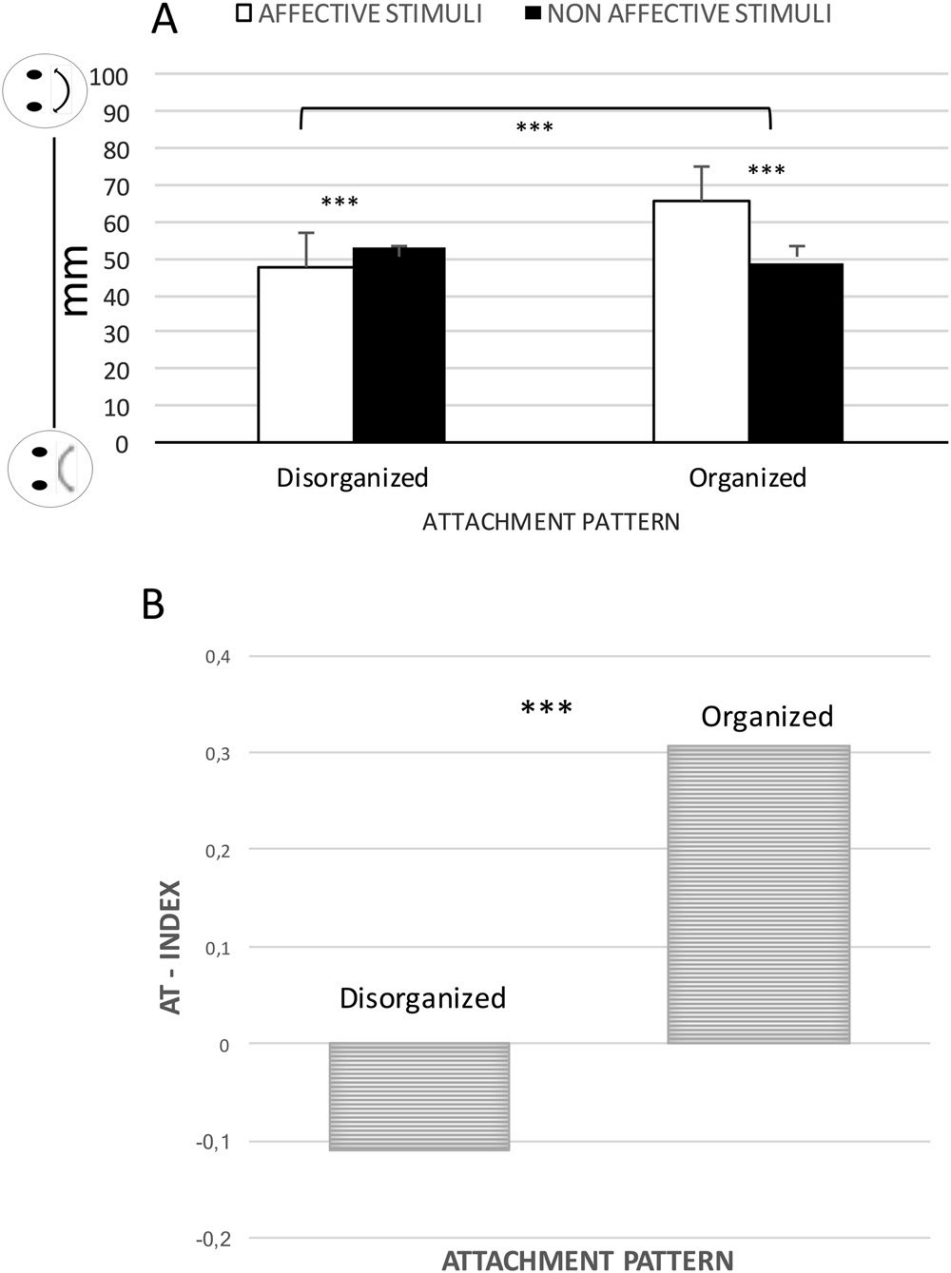
Panel A. Participants’ evaluations of the Affective stimulation and Non-affective stimulation. Panel B. Affective Touch Index. Participants’ general preference for Affective stimulation or Non-affective stimulation. Positive values represent the preference for Affective stimulation; negative values represent the preference for Non-affective stimulation.

The ANOVA with subjective perceptions of pleasantness as dependent variables, Stimulation (Affective – non-affective) as a within-subjects factor, and Attachment style (OA – DA) as a between-subjects factor, revealed a main effect of Group (*F*_1,628_ = 24.65; *p* = 0.001; Partial eta-squared = 0.038; observed power = 0.999) and of Stimulation (*F*_1,628_ = 11.73; *p* = 0.001; Partial eta-squared = 0.018; observed power = 0.929), and a Group-by-Stimulation interaction (*F*_1,628_ = 39.890; *p* = 0.001; Partial eta-squared = 0.060; observed power = 1.000). DA exhibited a lower evaluation of the pleasantness of touch; also, Affective stimulation was perceived as more pleasant than Non-affective stimulation. Interestingly, only OA perceived Affective stimulation more pleasant than Non-affective stimulation, as supported by post-hoc pairwise comparisons (see Fig. 1A).

#### Disorganized participants prefer Non-affective stimulation over pleasant stimulation

Also, DA’s general preference for Affective stimulation (namely, Affective Touch Index), was significantly lower than OA’s; indeed, they indicate a slight preference for the Non-affective stimulation over the Affective stimulation (*t*_(61)_ = 1.99; *p* < 0.05. *Cohen’s d* = 0,04) (see Fig. 1B).

Summarizing, we have found that people with a disorganized attachment pattern perceive the affective stimulations as less pleasant then people with organized attachment style. Moreover, the former exhibits a preference for the Non-affective stimulations rather than the pleasant ones.

Given the different responses between OA and DA in the evaluation of Affective Touch, the investigation of the neural correlates underlying affective and non-affective touches is compelling.

To this aim, we developed an fMRI study in which Affective stimulation and Non-affective stimulations were provided to a subgroup of individuals from Study 1, with organized or disorganized attachment.

### Study 2

#### Affective touch cerebral network

**Figure 2:**
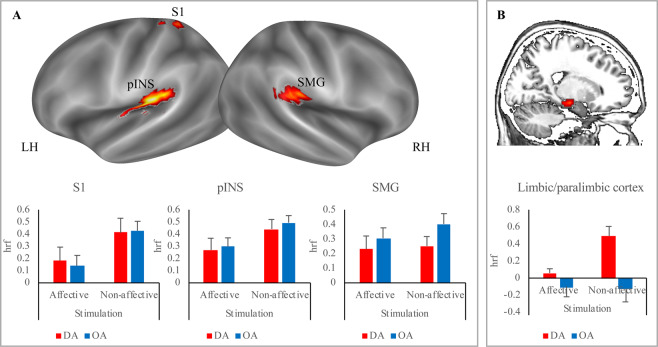
Imaging results. Brain areas involved in Affective and Non-affective stimulation, including posterior insula (pINS) and primary somatosensory cortex (S1) in the left hemisphere (LH) and supramarginal gyrus (SMG) and limbic/paralimbic cortex in the right hemisphere (RH). A main effect of the Stimulation was detected in the pINS and the S1 (Panel A). We found a significant Group-by-Stimulation interaction (Panel B) in the right limbic/paralimbic cortex with Non-affective stimulation yielded higher activation than Affective one in people with disorganized attachment (DA) but not in people with organized attachment (OA).

The first step of our analysis was aimed at providing a general picture of the brain areas involved in processing Affective stimulation and Non-affective stimulation. We found a network of areas encompassing the left posterior insula (pINS), the left primary somatosensory cortex (S1), the right supramarginal gyrus (SMG) and the right limbic/paralimbic cortex (Fig. 2A; Table 2). This last cluster extended from the paralimbic cortex, on the medial surface of the temporal lobe, to the midbrain, hippocampus and amygdala (see Fig. 2A).

**Table 2:** Brain network involved in processing Affective stimulation and Non-affective stimulation.

Region	Hemisphere	Label	cluster p(FWE)	peak F	peak p(unc)	k	x	y	z
*Posterior Insula*	*LH*	*PI*	*0.000*	*21.471*	*0.000*	144	*−*48	*−*28	19
				19.236	0.000		−39	−25	19
Limbic/paralimbic *cortex*	*RH*	limbic/paralimbic	*0.0*27	*13.423*	*0.000*	*27*	18	*−*19	*−*14
				8.793	0.000		12	−7	−14
*Supramarginal gyrus*	*RH*	*SMG*	*0.001*	*12.008*	*0.000*	53	51	*−*28	25
				6.874	0.000		63	−37	25
*Primary Somatosensory cortex*	*LH*	*S1*	*0.030*	*9.515*	*0.000*	26	*−*21	*−*40	61
				7.976	0.000		130	−31	64

Within this network, we found a main effect of the Stimulation in the pINS (*F*_1,20_ = 27.614; *p* = 0.000, partial eta square = 0.605, observed power = 0.999), the S1 (*F*_1,20_ = 45.559; *p* = 0.000; partial eta square = 0.717, observed power = 1.000) and the limbic/paralimbic cortex (*F*_1,20_ = 14.730; *p* = 0.001, partial eta square = 0.450, observed power = 0.952): these areas were more activated during Non-affective stimulation as compared with Affective stimulation (Fig. 2B).

#### Right limbic/paralimbic cortex is activated only by disorganized participants

A main effect of Group was detected in the right limbic/paralimbic cortex (*F*_1,20_ = 35.917; *p* = 0.000; partial eta square = 0.666, observed power = 1.000), with only DA showing activation of this region (Fig. 2B). Interestingly, we also found a significant Group-by-Stimulation interaction in the right limbic/paralimbic cortex (*F*_1,20_ = 17.138, *p* = 0.001, partial Eta square = 0.488; observed power = 0.974). Post hoc pairwise comparisons showed that Non-affective stimulation yielded higher activation than Affective stimulation in DA (*p* < 0.001; Bonferroni’s correction for multiple comparisons was applied), but not in OA (*p* = 0.814) (Fig. 2B).

Summing up the main findings, we found that only DA showed activation in the right limbic/paralimbic cortex, especially during Non-affective stimulation.

## Discussion

The first experience of newborns with the nearby environment occurs through touch and smell^36,37^. Through touch, babies discover the bond with their mothers, and they understand how to communicate their demands and needs. Good tactile caregiving improves the emotional, intellectual, and social functions of young children, suggesting the influence of positive and gentle touch from birth.

Our first study showed that adults with a disorganized attachment pattern do not perceive gentle touch as being a pleasant experience; rather, they regard Non-affective and Affective touch as neither pleasant nor unpleasant. In contrast, adults with an organized attachment pattern rate affective touch as being much more pleasant than Non-affective stimulation, significantly preferring caress-like touch. It is important to remember that the alterations observed in the evaluation of the Affective Touch cannot be related to basic touch dysfunctions since the discriminatory touch assessment through Two-Point discrimination test, Von Frey Monofilaments and thermal sensitivity showed no alterations in basic touch processing.

A possible explanation for this could be the different interpretation that the person with disorganized attachment makes of touch.

In fact, we know that the interpersonal functioning of people with disorganized attachment is characterized by a marked reduction of emotional regulation that leads the person to react with defensive behaviors even in situations of emotionally neutral interpersonal exchanges^38–40^.

We can therefore assume that, as for other interpersonal contexts, any tactile exchange (neutral or pleasant) is considered threatening and for this reason it is perceived as not pleasant. Further, the essential feature of disorganized attachment is the lack of a coherent strategy for interacting with and responding to a caregiver in times of stress^41^. From this perspective, when the person’s need for reassuring contact is not met regularly, he can develop confusing and erroneous beliefs regarding the value of physical contact.

A paradigmatic example is the need for self-harm (often expressed as cutting), exhibited by persons with BPD; notably, many BPD patients report analgesic phenomena during self-injury^42^, suggesting the possibility of top-down control of the pain. The literature suggests that the most prominent reason for self-harm in these patients is to reduce aversive inner tension to avoid unpleasant experiences and feelings, leading to temporary relief^43,44^.

Disorganized attachment has been related to personality disorders^45^. Several theorists have observed the similarities between the contradictory and disoriented behaviors of disorganized patterns and severe disturbances in attachment in individuals with personality disorders, particularly BPD^46–49^.

Our data on the reduction of tactile pleasure during Affective stimulation in disorganized participants could be interpreted as being akin to the analgesic/anesthetic phenomena that are observed during self-harm but in the hedonic domain of touch. The American academic Mary Main calls disorganized attachment “fear without solution”^50^. We speculate that the anesthetic phenomena that are observed during the experience of touch serve as an extreme attempt to temporarily solve the emotional state of alertness that is elicited by the continuous state of danger and fear.

Although to date there are no systematic studies investigating the physiological correlates of C-fibres in self harm behaviour, it is possible to assume that, in self-harm patients, this system may be altered. We know, in fact, that C-fibres are involved in the processing of stimuli that cause deep pain^51^ and that a specific class of C-fibres, namely the CT system, is devoted to process pleasant tactile stimuli (i.e. Affective Touch). Moreover, observations from healthy individuals suggested that CT-optimal stimulation appears to decrease pain perception^16,52,53^. It is possible to speculate that in self-injurious patients, these two functions of the C system (i.e. Affective Touch and nociception) may be partially overlapped, thus producing a feeling of pleasure even in the case of painful stimulation. Obviously, this proposal is little more than naive; to support this view there is a need for studies and research with specific hypotheses to be tested on a large number of patients.

Processing Affective stimulation and Non-affective stimulation, delivered to the top right forearm as tactile stimulation, relies on a wide network of areas, encompassing the left posterior insula, left primary somatosensory cortex, right supramarginal gyrus, and right limbic/paralimbic cortex. Within this network, the posterior insula, primary somatosensory cortex, and limbic/paralimbic cortex were more highly activated by Non-affective than Affective stimulation. In contrast, the right supramarginal gyrus was generally involved in Affective and Non-affective stimulation.

We found that only people with disorganized attachment activated the limbic/paralimbic cortex (including the amygdala, hippocampus, and midbrain) during Non-affective stimulation. These regions have been associated with a wide range of processes that involve motivated behaviors and emotion. Specifically, the amygdala has been hypothesized to represent valence, by processing the general appetitive/aversive affective characteristics of stimuli. Accordingly, neurophysiological findings show that amygdalar neurons respond differentially between stimuli with positive and negative affective significance, thus providing a representation that is useful for coordinating physiological, behavioral, and cognitive responses in an affective/emotional context^54^. It has also been associated with the processing of unpleasant emotions that are elicited by affective pictures^55^, fear conditioning^56–59^, fear extinction, and reinforcer devaluation^54^. Remarkably, it has been recently found that people with interpersonal traumatization, who strongly disliked interpersonal skin-to-skin stroking, showed reduced hippocampal activation during interpersonal touch^23^. Finding of aberrant hippocampal activation, even if in the opposite direction we found in our study, support the idea that this region hosts aberrant mechanisms of touch processing.

Also, the hippocampus and amygdala are involved in novelty detection, contributing to various extents—both regions are process novel common stimuli, but the amygdala is also activated by unusual stimuli^60^. Notably, people with unresolved-disorganized attachment show aberrant resting state functional connectivity in the amygdala^61^. Current evidence has demonstrated that individuals with disorganized attachment undergo greater activation of the limbic/paralimbic areas, which process the valence of stimuli to coordinate physiological, behavioral, and cognitive responses in affective/emotional contexts, consistent with our assumptions on the involvement of these areas in processing general emotional arousal.

Romantic and maternal love have been found to suppress activity in the brain regions that are associated with the processing of negative emotions, including the amygdala and medial temporal lobe^62^. In 2 earlier studies, the Adult Attachment Projective Picture System was shown to activate the amygdala and paralimbic cortex^63,64^. Based on these findings and our results, the limbic/paralimbic cortex—which we found to be more highly activated in people with disorganized attachment—might be the key node that subtends the abnormal alertness to touch. However, this alteration is due primarily to Non-affective stimulation.

Two possible explanations should be considered. With regard to people with disorganized attachment who failed to recognize the pleasantness of gentle touch (see the results of Study 1), they never experienced affective stimulation to be pleasant, unable to note any difference between Non-affective and Affective stimulation, which is otherwise detected in people with organized attachment. Regardless of the frequency of stimulation (Affective/Non-affective), people with disorganized attachment never perceive the touch as being pleasant. Thus, as expected, the difference that we observed is attributed to Non-affective stimulation, which is not pleasant.

In the alternate possibility, Non-affective stimulation occurs at 30 cm/s (non-CT-optimal velocity), faster than Affective stimulation, which proceeds at 3 cm/s (CT-optimal velocity). It is possible that people with disorganized attachment, who rated Affective and Non-affective stimulation similarly, experienced higher activation in this region, because this more rapid stimulation inevitably implies more brushes than affective touch and is thus more alerting. Consistently, Non-affective stimulation also effects greater activation in brain areas that are devoted to somatosensory perception (eg, S1 and pINS). In light of this evidence, it is possible that only Non-affective (ie, more frequent) stimulation produces a response in these individuals. This explanation mirrors the need for more intense stimulation (eg, self-cutting and other self-harm behaviors), as frequently reported in patients with personality disorders, such as borderline personality disorder, that are characterized by unsolved attachment patterns^65^. Notably, patients with borderline personality disorder and disorganized attachment undergo greater activation in the amygdala in response to the Adult Attachment Projective Picture System^65^. Collectively, this evidence has possible clinical applications, meriting further study.

It should be noted that, unlike what was found in the literature (i.e. Morrison, 2016), in our study we found activation of the posterior insula also during CT-suboptimal stimulation. This data, if on the one hand may be due to the different experimental paradigm used in the various studies, on the other hand indicates the need to further investigate the function of the posterior insula in the processing of AT.

One of the limitations of this report is the small number of participants in the fMRI study. Many participants with disorganized attachment refused to participate in the fMRI study or withdrew. Notwithstanding, the power that we calculated (observed power >0.952) strongly supports our results, despite the small sample size. However, the low number of participants did not allow us to test possible sex-related effects, which future studies should determine. Also, whether abnormal activation in the limbic/paralimbic cortex is strictly linked to c-fibers, especially by stimulating non-CT glabrous skin, such as the palm, should be tested^66^.

Our findings have possible applications in psychopathology. Specifically, personality disorders—for example, borderline personality disorder—might be associated with disrupted affective processing. In this light, the examination of emotional appraisal during painful and unpleasant stimulation might yield compelling results.

## Methods

### Study 1

#### Participants

63 healthy Caucasian subjects (31 females and 32 males), were recruited from the general population by word of mouth and the use of flyers distributed in a commercial area of downtown (e.g., bookshops, cafeterias, and public library). Exclusion criteria, assessed during a pre-screening semi-structured interview, were: diagnosis of neurological disease, substance abuse/dependence and pregnancy or childbirth within the last 12 months.

#### Procedure

Participants were first evaluated for possible inclusion in the study by means of an informal interview aiming to get a thorough acquaintance. Then, they were invited to arrange an appointment with the researcher for the experimental meeting and all of them were evaluated in a single session lasting about 75 minutes. The protocol consisted in four steps: the Adult Attachment Interview (AAI), the drawing up of two psychological scales (the Personality Inventory for DSM-5-PID-5; and the Symptom Checklist-90-R), the tactile assessment (Von Frey Monofilaments for the assessment of the tactile sensitivity; Two-Point Discrimination test (2PD) for tactile acuity and thermal threshold for the thermal sensitivity) and the Affective Touch experiment. The AAI was always conducted first, whereas the remaining assessments were balanced between participants; thus, 50% of participants received the experimental tactile procedure before the psychological scales, and the remaining 50% took these latter before the tactile session. For a full description of the AAI and the psychological scales, see Supplementary material.

The protocol was approved by the local Ethics Committee (Comitato Etico Presso la Fondazione Santa Lucia di Roma) and conformed to The Code of Ethics of the World Medical Association (Declaration of Helsinki), as printed in the British Medical Journal (July 18, 1964). All participants provided written informed consent.

#### Tactile assessment

Von Frey Monofilaments. The Von Frey test is a classical measure of sensitivity to tactile pressure that is used for diagnostic and research purposes (North Coast Medical, Inc., Morgan Hill, CA, USA).

Two-Point discrimination test. Tactile Acuity was estimated using the two-point discrimination thresholds with an adjustable aesthesiometer (Med Core, St. Louis, MO, USA) with 2 separate tips.

Thermal sensitivity. Caloric sensitivity was tested using a TSA II device (MEDOC Inc., Ramat Ishai, Israel). Baseline temperature was always set to 32 °C and then successively heated with a ramp rate of 1 °C per second.

All tactile assessments were carried out in the hairy skin of the dominant forearm”.

Extended description of all the tactile measures in the supplementary section

#### Affective touch experiment

In the experimental session, participants received Affective and Non-affective tactile stimulations over their upper dominant forearm (hand dominance was assessed by the Edinburgh Handedness Inventory^67^). As in previous studies^34^ stimuli were delivered manually by the same experimenter, who was trained to apply the strokes with constant force and velocity. Participants were instructed to sit still with their eyes closed during the stimulation and to focus on the tactile sensation. Then the participants were asked to rate their subjective perception of pleasantness for each stroke on a visual analogue scale (VAS), with unpleasant (sad face) and pleasant (smiley face) as endpoints, ranging from 0 to 100 (millimetres). Before starting the experimental protocol, the subjects were familiarized with the stimuli. First, they were asked to undergo to 10 warm-up trials and then they were asked to indicate on various points on the VAS (e.g. ‘totally unpleasant,’ ‘pretty unpleasant,’ ‘average,’ ‘pretty pleasant,’ ‘totally pleasant’) to evaluate their comprehension of the instructions.

Extended description of the experimental setup in the supplementary section. (Fig. S1).

Stimuli. Tactile stimulation of the right dominant dorsal forearm was delivered manually with a soft goat’s hairbrush (0.5 cm wide, 3 cm long) at 2 velocities—CT-optimal (3 cm/s) and non-CT-optimal (30 cm/s). CT-optimal velocity indicates the optimal speed of stimulation through which people usually perceive a pleasant sensation; non-CT-optimal velocity identifies the speed of stimulation through which people usually perceive a neutral tactile sensation (no pleasant nor unpleasant). 20 CT-optimal and 40 non-CT-optimal stimulations were delivered to each subject in a pseudorandomized order and the subjects’ responses (in mm) were used in the analyses. In order to simplify the reading of the results, the term “CT-optimal stimulation” was relabelled Affective stimulation and the term “non-CT-optimal stimulations” was relabelled Non-affective stimulations. Also, the Affective Touch Index was calculated for each participant. As proposed by Croy *et al*.^68^ the Affective Touch Index provides the individual preference for Affective or Non-affective stimulations and is defined as the individual difference in pleasantness rating between the CT-optimal (i.e., 3 cm/s) and non-CT-optimal stroking velocities (i.e., 30 cm/s), weighted by the overall pleasantness of the touch. Positive values for the Affective Touch Index indicate a preference for the Affective over Non-affective stroking velocity.

#### Experimental protocol

The tactile protocol was divided into 2 halves separated by a break to prevent fatigue and tactile habituation. A grid was drawn on the hairy skin of the long axis of the participants’ dominant forearm to guide the experimenters during the stimulation. To further minimize habituation, tactile stimulation was alternated between the rows that were drawn on the forearm (from proximal to distal).

#### Statistical analyses

Data processing was performed using SPSS (IBM). Partial eta-squared (ηp^2^) and Cohen’s d were calculated to quantify the effect sizes of all comparisons. On the bases of the AAI, the entire sample has been split into two groups, namely Organized Attachment (OA; N = 46) and Disorganized Attachment (DA; N = 17).

To evaluate group differences in the demographics and clinical variables of the study, two sample t-tests were computed on age, education, SCL-90-R, PID-5, Von Frey, Thermal sensitivity and 2PD. Chi-square comparison was conducted to test for pre-existing differences in sex distribution.

#### ANOVA

To examine the differences between OA and DA, in the perception of Affective Touch, we ran a mixed factorial 2-by-2 ANOVA, with Group (OA vs. DA) as between factor and Stimulation (Affective vs. Non-affective) as repeated measure.

### Study 2

#### Participants

We initially enrolled 12 adults (mean age 28.33, SD 6.14; 5 women) with organized attachment (OA) and 12 with disorganized attachment (DA) from the study 1; however, among this latter group, only 8 participants successfully completed the fMRI study (mean age 37.25, SD 13.41; 5 women): indeed, 4 individuals refused to perform the entire fMRI acquisition due to high level of anxiety and low compliance with experimental setup.

#### Stimuli

Stimuli consisted in manual strokes with a 4-cm wide watercolour brush applied on the hairy skin of the dorsal forearm. Continuous brushing (back and forth) was applied to the right forearm at CT optimal velocity (3 cm/s) or non-CT optimal velocity (30 cm/s), namely Affective and Non-affective stimulation respectively^69^.

#### Procedure

Participants were asked to close their eyes and focus on the touch they experienced. 15 cm of the forearm were marked to control for the length of stimulated skin in each participant. Three trained experimenters (P.Z., G.C. and G.T.) administered the stimuli during fMRI. The 2 experimental conditions (Affective stimulation vs. Non-affective stimulation) were presented in separate blocks in 4 consecutive fMRI runs. Order of blocks was balanced following an A-B-B-A schema. Each run consisted of 10 blocks (half were Affective stimulations) and started with a 4 s period of rest, during which the experimenter got ready to provide the tactile stimulation depicted on the screen. Then, tactile stimulation was provided for 12 s, followed by 12 s of rest.

#### Image acquisition

A Philips Achieva scanner operating at 3 T and equipped for echo-planar imaging was used to acquire functional magnetic resonance images using 32-channels SENSE head coil. Head movements were minimized with mild restraint and cushioning. Functional MRI images were acquired for the entire cortex using blood-oxygen-level-dependent (BOLD) contrast imaging (38 slices, in-plane resolution = 2.5 × 2.5 mm, slice thickness = 4 mm, repetition time (TR) = 2 s, echo time (TE) = 30 ms, flip angle = 77 deg). For each scan 122 fMR volumes were acquired. We also acquired a three-dimensional high-resolution T1-weighted structural image for each subject (parameters: 342 slices, in-plane resolution = 0.5 × 0.5 mm, slice thickness = 0.5 mm, TR = 2 s, TE = 5.75 ms, flip angle = 8 deg).

#### Image analysis

Image analysis was performed using SPM12 (http://www.fil.ion.ucl.ac.uk/spm).

As a preliminary step of our analyses we identified the neural network underlying Affective and Non-affective tactile stimulation. To this aim we ran a full factorial design, with Group (OA vs. DA) and Stimulation (Affective vs. Non-affective) as factors. Thus, we computed an F-omnibus contrast of all conditions (activation only) and the resulting statistical parametrical maps were thresholded at p < 0.05 at the cluster level using Family Wise Error correction (FWE), after forming clusters of adjacent voxels surviving a threshold of p < 0.001 uncorrected. This orthogonal contrast allowed us to derive clusters of regions to be used in subsequent steps, increasing the sensitivity of the analyses, without incurring in the risk of circularity^70,71^. For each subject and region, we extracted average parameter estimates of HRF in each experimental condition (i.e. Stimulation), across all voxels in the region. Regionally averaged data were then analysed with mixed factorial ANOVA, with the same factorial structure employed in the ANOVA of the Study 1 – namely, Group (OA vs. DA) as between factor and Stimulation (Affective vs. Non-affective) as within factor.

Extended description of image acquisition protocol and preprocessing details is provided in the supplementary materials.

## Supplementary information

Supplementary.
