# Supplementary material for: Disorganized Attachment pattern affects the perception of Affective Touch
Source: Sci Rep. 2020 Jun 15;10:9658. doi: 10.1038/s41598-020-66606-5 (PMC7295781; doi:10.1038/s41598-020-66606-5)
Supplement: Supplementary file 1 — Supplementary. [file 41598_2020_66606_MOESM1_ESM.pdf]

Supplementary Information

Disorganized Attachment pattern affects the perception of Affective Touch

Grazia Fernanda Spitoni*[1,2], Pietro Zingaretti[3], Guido Giovanardi[4], Gabriella Antonucci[2,5], Gaspare Galati[2,5], Vittorio Lingiardi[1], Gianluca Cruciani[6], Giulia Titone[7], Maddalena Boccia[2,5]

[1] *Department of Dynamic and Clinic Psychology, Sapienza University of Rome, Rome, Italy*

[2] *Cognitive and Motor Rehabilitation and Neuroimaging Unit, Santa Lucia Foundation (IRCCS Fondazione Santa Lucia), Rome, Italy*

[3] *Villa von Siebenthal Hospital, Genzano di Roma, Italy*

[4] *Department of Psychology, University of Campania, Luigi Vanvitelli, Caserta, Italy*

[5] *Department of Psychology, Sapienza University of Rome, Rome, Italy*

[6] *Department of Psychology, PhD Program in Behavioral Neuroscience, Sapienza University of Rome, Rome, Italy*

[7] *Utrecht University, Faculty of Social and Behavioural Sciences, Experimental Psychology, Utrecht, The Netherlands*

**Corresponding author:* Grazia Fernanda Spitoni, via dei Marsi, 78, 00185, Department of Psychology–Sapienza University of Rome, Rome, Italy. E-mail address: grazia.spitoni@uniroma1.it

Supplementary Methods

Study 1

Semi-structured interview

1 Generic information:

- How did you find out about the study?
- Has anyone you know already done the experiment?
- Have you done other experiments in the past in this or in other laboratories?

2 Personal information

- How old are you?
- Would you mind doing a long interview with a clinical psychologist about your past and family relationships?
- If it did, would you be willing to do an MRI at our hospital?

3 Health info

- Can you rule out being pregnant at this stage? (women only)
- Have you ever suffered from skin problems?
- Have you ever suffered from neurological or psychiatric problems? (in case of need you can give examples of psychiatric or neurological disorders)
- Are you currently or in the last 12 months on any medications?

Would you like to be contacted in case of any other experiments?

Measures

Adult Attachment Interview

The Adult Attachment Interview (AAI) [1] is a semi-structured, clinical interview designed to assess an individual's current state of mind with respect to past caregiver–child attachment-related experiences [2]. Interpretations of the adult attachment categories do not rely on the

assumption that they represent veridical accounts of early childhood experience; rather, transcripts of the interviews are coded by trained raters according to how coherently people recall their past experiences. The individual's strategy during the AAI (e.g., derogating or minimizing of attachment vs. valuing and rendering a balanced, coherent narrative despite positivity or negativity of actual experience) is supposed to reflect the quality or security of one's current state of mind with respect to attachment [2]. Typically, one out of three possible main classifications is assigned to the most prominent state of mind throughout the interview as a whole: secure/autonomous (F), insecure-dismissing (Ds), or insecure-preoccupied (E), of which secure/autonomous is considered the most beneficial. Furthermore, when present, discussions of experiences of loss, abuse, or other potential trauma are scored for disorientation in reasoning or discourse and, when sufficiently marked, may lead to a primary classification of a disorganized/unresolved (Ud) state of mind. In such a case, a secondary (organized) classification of secure/autonomous, insecure/dismissing, or insecure/preoccupied is assigned for the remaining narrative. Interviews in which a singular organized state of mind cannot be identified (e.g., because marked indications of several states of mind are present) are coded as cannot classify (CC) [3]. The AAIs were transcribed verbatim, and identifying information was removed prior to coding. To assess individual differences in attachment, transcripts were coded by a certified AAI coder, who had achieved greater than 80% agreement with on the official reliability test.

Psychological scales

Personality Inventory for DSM-5. The personality Inventory for DSM-5 (PID-5) [4] is a 220-item self-report measure of the DSM-5 alternative personality disorder model traits. The PID-5 measures 25 personality traits that can be organized into five overarching domains (i.e., negative affect vs. emotional stability, detachment vs. extraversion, psychoticism vs. lucidity, antagonism vs. agreeableness, and disinhibition vs. conscientiousness). Each trait is assessed by 4 to 14 items and evidence from non-clinical samples indicated that the PID-5 latent trait domain structures were concordant with Five Factor Model traits [5] and demonstrated good convergence with well-established personality trait measures [6-9].

Symptom Checklist-90-R. The Symptom Checklist-90-R (SCL-90-R) [10] is a self-report questionnaire composed of 90 items exploring the frequency of several psychological symptoms in the last week. Respondents are asked to answer on a 5-point Likert scale ranging from 0 (not at all) to 4 (extremely). The nine clinical subscales are Somatization, Obsessive-Compulsive, Interpersonal Sensitivity, Depression, Anxiety, Phobic Anxiety, Psychoticism, Paranoid Ideation, and Hostility. The global indices include the Global Severity Index (GSI), the Positive Symptom Distress Index (PSDI), and the Positive Symptom Index (PSI). Overall, the SCL-90-R subscales have demonstrated excellent internal consistency (.77 to .90) and test–retest reliability (.78 to .90) [11].

Tactile assessment

Von Frey Monofilaments. In this test, the tip of a fiber with a specific weight (from 0.008 to 300 g) is pressed against the skin at right angles. The force of application increases as the researcher advances the probe until the fiber bends. In this study, the participants were instructed to sit still with their eyes closed during the procedure and focus on the tactile sensation. The procedure was repeated using various weights of fibers, forming an ascending and descending staircase. At each

level of the staircase, 10 actual stimulations and 5 catch trials (a total of 15 stimulations) were presented. In each trial, the experimenter asked the participants whether they felt the stimulus, to which they had to respond verbally. The threshold was established at the level when the subjects reported 6 of 10 stimuli correctly.

Two-Point discrimination test. Stimuli were delivered manually to the dominant forearm. Participants were instructed to sit still with their eyes closed during the procedure discriminate between single and double taps, responding verbally. In this procedure, double or single taps were administered randomly. Only double taps were used to calculate the threshold. The separation between the 2 starting points was 1 and 5 cm in the ascending and descending modes, respectively. The separation was then decreased by 0.5 cm after each correct response. When an error was made, the separation rose by 0.5 cm. The participants' threshold was derived from the minimum distance that was perceived between the 2 points 5 times consecutively.

Thermal sensitivity. A warming cylinder with 1,5 cm of diameter was placed on the dorsal side of the right forearm and participants were instructed to indicate verbally as soon as the heat became intolerable. To prevent tissue damage, maximum duration of the heat exposure was set at 40sec. The assessment was administered five times and the average of the measurements was used in the analysis.

Study 2

Image Acquisition

A Philips Achieva scanner operating at 3T and equipped for echo-planar imaging was used to acquire functional magnetic resonance images using 32-channels SENSE head coil. Head movements were minimized with mild restraint and cushioning. Functional MRI images were acquired for the entire cortex using blood-oxygen-level-dependent (BOLD) contrast imaging (38 slices, in-plane resolution = 2.5 x 2.5 mm, slice thickness = 4 mm, repetition time (TR) = 2 s, echo time (TE) = 30 ms, flip angle = 77 deg). For each scan 122 fMR volumes were acquired. We also

acquired a three-dimensional high-resolution T1-weighted structural image for each subject (parameters: 342 slices, in-plane resolution = 0.5 x 0.5 mm, slice thickness = 0.5 mm, TR = 2 s, TE = 5.75 ms, flip angle = 8 deg).

Image analysis

The first four volumes of each run were discarded to allow for T1 equilibration. All images were corrected for head movements (realignment) using the first volume as reference. The images of each participant were then coregistered onto their T1 image. Coregistered images were then normalized to the standard MNI-152 EPI template using the mean realigned image as a source. Images were then spatially smoothed using a 6-mm full-width half-maximum isotropic Gaussian kernel. Functional images were analyzed for each subject separately on a voxel-by-voxel basis according to the general linear model (GLM). Neural activation during the blocks was modeled as a boxcar function spanning the whole duration of the blocks and convolved with a canonical hemodynamic response function, chosen to represent the relationship between neuronal activation and blood oxygenation [12]. Separate regressors were included for Affective vs. Neutral. Inter-block intervals were also modeled in relation to the nature of the previous block (Affective-rest vs. Neutral-rest). Group analysis was performed on estimated images that resulted from the individual models of each condition (Affective vs. Neutral) compared with its baseline (Affective-rest vs. Neutral-rest), treating subject as a random factor.

Supplementary Figures

Figure S1: Experimental setup. Tactile stimulation of the right dominant dorsal forearm was delivered manually with a soft goat's hair brush (2,5 cm wide, 3 cm long). In order to guarantee the highest control of the stimulation, the experimenter wore earphones and was skilled to use acoustic signals to stimulate at the precise velocity and in the correct temporal sequence. Earphones were triggered by a remote computerized metronome, previously programmed to provide the exact velocities. To guide the experimenters during the stimulation, a grid was drawn on the hairy skin of the long axis of the participants' dominant forearm. To minimize CT habituation, four different areas of the forearm delimitated by the grid were stroke (two laterals and two medials; stimulation direction: from proximal to distal).

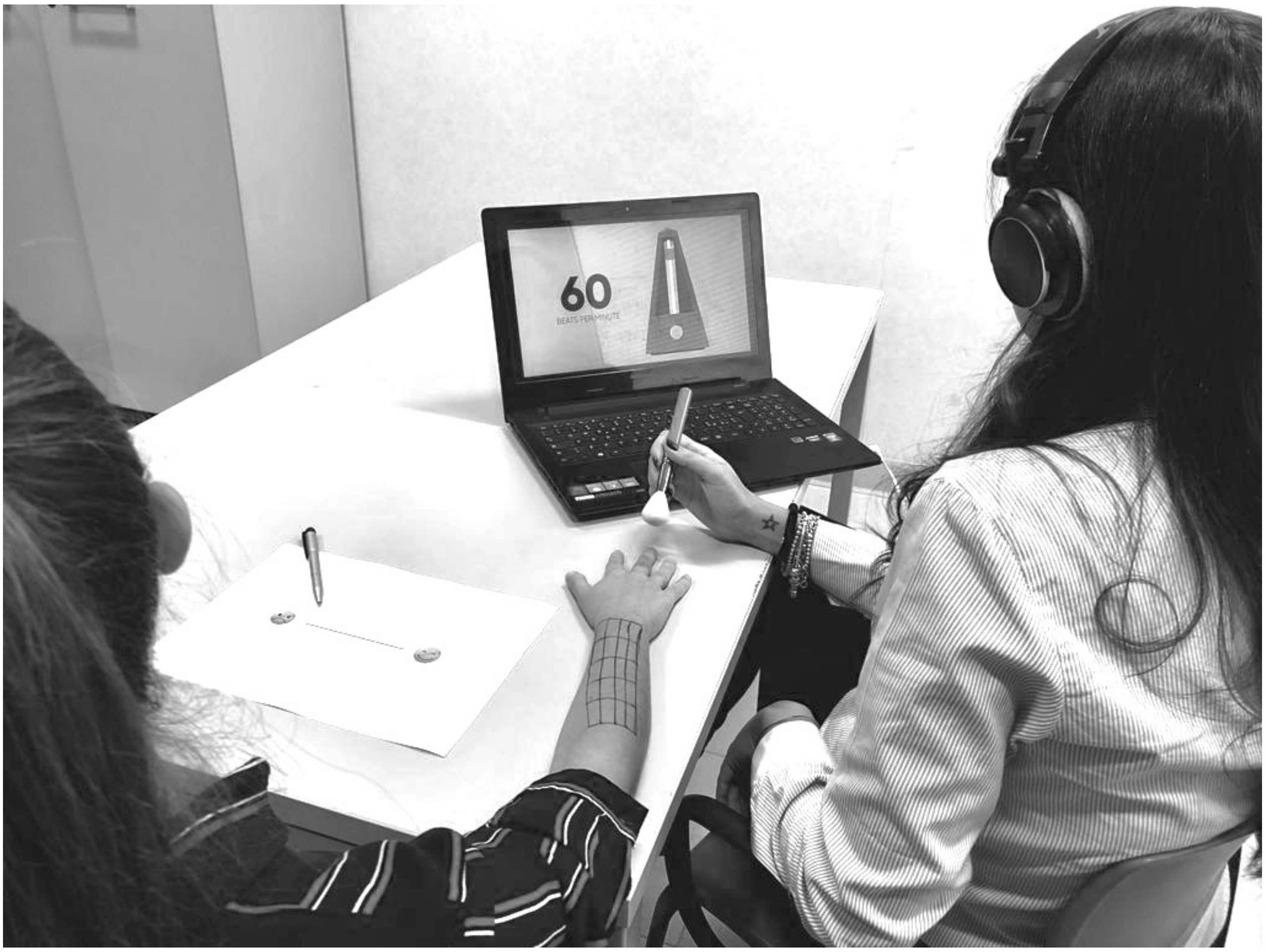

Supplementary References

1. George, C., Kaplan, N., & Main, M. *Adult attachment interview. Unpublished manuscript, (3rd ed.)* (Department of Psychology, University of California, Berkley, 1996).

2. Hesse, E. The Adult Attachment Interview: Protocol, method of analysis, and empirical studies in *Handbook of attachment: vol. 2. Theory, research, and clinical applications (2nd ed.)* (Eds. Cassidy, J., & Shaver, P. R.) 552-598 (New York: Guilford Press, 2008).

3. Reijman, S. et al. Attachment representations and autonomic regulation in maltreating and nonmaltreating mothers. *Dev. Psychopathol.* **29**(3), 1075-1087. (2017).

4. Krueger, R. F., Derringer, J., Markon, K. E., Watson, D., & Skodol, A. E. Initial construction of a maladaptive personality trait model and inventory for DSM-5. *Psychol. Med.* **42**(9), 1879-1890 (2012).

5. Thomas, K. M. et al. The convergent structure of DSM-5 personality trait facets and Five-Factor model trait domains. *Assessment* **20**(3), 308-311 (2013).

6. Anderson, J. L. et al. On the convergence between PSY-5 domains and PID-5 domains and facets: Implications for assessment of DSM-5 personality traits. *Assessment* **20**(3), 286-294 (2013).

7. Ashton, M. C., Lee, K., de Vries, R. E., Hendrickse, J., & Born, M. P. The maladaptive personality traits of the Personality Inventory for DSM-5 (PID-5) in relation to the HEXACO personality factors and schizotypy/dissociation. *J. Pers. Disord.* **26**(5), 641-659 (2012).

8. Fossati, A., Krueger, R. F., Markon, K. E., Borroni, S., & Maffei, C. Reliability and validity of the Personality Inventory for DSM-5 (PID-5) predicting DSM-IV personality disorders and psychopathy in community-dwelling Italian adults. *Assessment* **20**(6), 689-708 (2013).

9. Wright A. G. C. et al. The hierarchical structure of DSM-5 pathological personality traits. *J. Abnorm. Psychol.* **121**(4), 951 (2012).

10. Derogatis, L. R. *The symptom checklist 90-R: administration, scoring and procedures manual (3rd ed.)*. (Minneapolis, MN: National Computing Systems, 1994).

11. Payne, R. W. Review of the SCL-90-R. *Mental measurements yearbook*, 1325-1326 (1985).

12. Friston, K. J. et al. Event-related fMRI: characterizing differential responses. *Neuroimage* **7**(1), 30-40 (1998).
